# Factors influencing resilience of parents with children with neurodevelopmental disorders: The role of structural language, social cognition, and social support

**DOI:** 10.3389/fpsyt.2022.886590

**Published:** 2022-09-08

**Authors:** Raquel Flores-Buils, Clara Andrés-Roqueta

**Affiliations:** Department of Developmental, Educational Social and Methodological Psychology, Universitat Jaume I, Castellón de la Plana, Spain

**Keywords:** parental resilience, neurodevelopmental disorders (NDD), autistic spectrum disorders (ASD), attention-deficit/hyperactivity disorder (ADHD), developmental language disorder (DLD), social cognition, structural language, social support

## Abstract

**Background:**

Resilience allows a more positive coping and improves parents' wellbeing when they face a difficult situation like having a child with a neurodevelopmental disorder (NDD). We aim to analyze the development of resilience in parents of children with different NDD (ASD, DLD and ADHD) with different levels of structural language and social cognition, as well as the social support available for their families, and compare it to children with typical development (TD).

**Method:**

We analyzed the level of resilience of 156 parents, 73 with children with TD and 73 with three different NDD, taking into account variables such as age, structural language (receptive grammar) and social cognition (emotional understanding) of the children, and also the type of social support available to them.

**Results:**

Children with DLD and ASD showed lower receptive grammar and emotional comprehension skills, although only parents of children with ASD obtained better resilience scores. Moreover, age of children and formal support variables predicted the resilience of the parents according to the type of NDD.

**Discussion:**

The severity of social cognition and structural language difficulties of children with NDD and the fact of having support from professionals and family associations have a significant influence on the development of parental resilience.

## Introduction

Having a child with a disability becomes an adverse situation for parents because it involves many physical and emotional demands, sacrifices to meet their needs, or living with the insecurity of having an uncertain future (1).

In this regard, when raising children with neurodevelopmental disorders (NDD)—such as autistic spectrum disorder (ASD), attention-deficit/hyperactivity disorder (ADHD) or developmental language disorder (DLD)—who face social communication and behavioral difficulties to a greater or lesser extent, parents experience complex everyday situations that often lead to higher levels of stress (2), greater depressive symptoms (3), and sometimes poorer quality of life in comparison to parents with typically developing children (TD) (4).

The literature shows different studies that relate inappropriate behaviors of children with NDD to parental mental health (5, 6), but these studies were mostly conducted with parents of children with ASD (7). Therefore, the impact of behaviors characteristic of disorders such as ADHD or DLD on the mental health of their parents is less known.

Existing empirical evidence comparing the impact on the mental health of parents with children with ASD with other difficulties such as Down Syndrome (DS) or Intellectual Disability (ID), report large differences due to the type of behaviors specific to each disorder. Authors conclude that ASD is the disorder that produces the most parental stress and also that the severity of emotional and behavioral problems of these children are associated with the higher levels of stress of their parents (8, 9).

Given this fact, recent studies have focused on exploring factors that protect parents during the care and upbringing of an autistic child, stressing the importance of resilience skills. Resilience refers to an adaptive response that is activated when a person faces difficult conditions (10) (such as having a child with difficulties), facing the situation in a positive way, and coming out of it strengthened (11). Having resilient skills does not imply not experiencing these situations as adverse, but knowing how to handle, cope with and overcome them (12). The main constructs of resilience theory risk factors (those that predispose people to physical and mental health problems because they affect the way a person adapts to stress), protective factors (those that promote resilience by reducing the effects of risk and the negative reaction to it), and resilience indicators (internal aspects such as self-efficacy, acceptance, sense of coherence or optimism) (13). According to resilience theory, an individual's resilience is determined by the balance between these factors in the face of adversity (14).

Consequently, resilience significantly reduces stress (15, 16) and improves parental mental health (17), including suffering less depression (18), perceiving greater psychological wellbeing (19) and greater life satisfaction (20).

In this sense, literature specifies that risk factors for caregivers of individuals with ASD include severity of symptoms (21–23), poor family relationship (24, 25), and having more than one child with ASD (26). Several studies relate the severity of ASD symptoms (including social communication aspects) to lower parental psychological wellbeing, lower life satisfaction, and a higher prevalence of depression (25, 26). Particularly, it was seen that emotional, communication and behavioral problems of the children increased the mental health problems of their mothers (27).

In parallel, other studies show that social communication is related with development of structural language and social cognition skills not only in children with ASD (28), but also in children with DLD (29) and ADHD (30), who usually differ from children with ASD in the degree of their pragmatic language impairments. In this regard, although structural language (specially grammar) and social cognition abilities (including theory of mind and emotion understanding) are prerequisites of social communication skills, there are few studies that analyse whether they are associated with parental resilience in children with different levels of ASD or with other NDD (31).

Within relatives of children with ASD, protective factors of parental resilience are the age of the child (26), the social support of the family (32, 33), and other aspects such as religious beliefs and spirituality (34). In this sense, behaviors inappropriate to age are related to the perceived stress of parents of children with ASD (28) and to parenting burden (25). Moreover, social support has been shown to alleviate the impact of children's NDD symptom severity on parents' mental health, improving their wellbeing and reducing anxiety and depressive symptoms (35, 36).

However, few studies have compared the level of resilience in parents with different NDD who present social communication problems in a greater or a lesser degree.

In parallel, the paper of social support for the parents of children with NDD has been raised. First, formal support received from a professional is a source of informational and instrumental support, which provides resources for the whole family (such as attending a psychoeducational intervention or occupational therapy center) (20). Second, informal support includes attending family associations or activities with other families provides emotional support and a space for unwind (such as being part of an association of parents of children with a specific disorder, parenting schools or clubs that organize activities for parents) (37). And third, family support can act as a protective factor by having support for the care of children with NDD, as the presence of family members who assist in the care of the child with problems reduces the burden and stress on parents (38).

However, mixed evidences have been found regarding the availability of social support, and some of them point out that this social support is not related to parent's adaptation and resilience (39), which may be due to a failure to account for different types of social support. In this sense, although most existent studies deal with social support in a comprehensive manner, different types of social support can be distinguished (40).

So, as pointed above, since most of the studies that analyze mental health and resilience development of parents of children with NDD are focused on children with ASD, it is considered necessary to make a cross-disorder comparison of these variables including other children with NDD with mild but existent social cognition and language difficulties that could lead to inappropriate social and emotional behaviors, such as children with ADHD and DLD.

Consequently, this comparison will allow us to understand which factors influence the development of parental resilience of a child with NDD, and therefore, to establish guidelines to train parents of children with NDD to develop strategies to modify those behaviors of their children that generate stressful situations. Furthermore, considering the positive influence of resilience on the wellbeing of parents with children with NDD, this comparison will allow us to establish the type of supports required by each group as well. In this sense, it will help us to clarify the weight of social supports in the development of resilience, and will enable us to know better how different types of social support influence the development of resilience in parents with children with different NDD. Finally, knowing both the characteristics of the children and the contextual aspects that influence in the development of resilience will allow us to specify what aspects need to be worked on to foster resilient skills in their parents. This is to provide them of skills to better adapt themselves to the situation of having a child with NDD, what will lead them to improve their wellbeing and mental health.

Therefore, the overall aim of the present study is to examine the development of resilience of parents of children with different NDD, and to find out the influence of age, structural language and social cognition skills of their children, as well as the influence of different types of social support in the family (formal, informal and family support).

Accordingly, the following hypotheses are proposed:

- Parents of children with NDD will present higher scores on all factors that make up resilience (persistence, tenacity and self-efficacy; control under pressure; adaptability and support networks; control and purpose; spirituality) in comparison to parents of children with TD.
- Within children with NDD, parents of children with ASD (and subsequent more social communication problems), will present a higher level of resilience than parents of children with ADHD and DLD.
- Among personal features of children with NDD, factors such as age, structural language and social cognition will predict the resilience of parents of children with any type of NDD. Among contextual factors, formal support is the factor that will most influence the resilience of parents of children with any type of NDD.

## Methods

### Participants

A non-probabilistic convenience sampling method was used for the present study. A total of 156 children attending different ordinary schools in Spain together with their 156 parents of children took part of the sample: 73 were parents of 73 children with a diagnosed NDD (clinical group), and 73 were parents of 73 children with TD (control group). Both samples came from the same context and ordinary public schools, so all of the participant families had a similar average socio-economic status.

All the selected children with NDD (57 boys and 16 girls) had an updated diagnosis at the time of the investigation and had been previously diagnosed by school psychologists following the regional protocols and meeting the DSM-5 criteria for the assessment of these disorders with standardized common specific measures. They were all aged between 5 and 12 years (M = 107.81 months; SD = 26.83; range 60–155). This clinical group (NDD) was organized intro three subgroups:

- 36 parents and 36 children with ASD (M = 106.78 months; SD = 25.41; range 60–154).
- 20 parents and 20 children with ADHD (M = 117.05 months; SD = 22.88; range 74–155).
- 17 parents and 17 children with DLD (M = 99.12 months; SD = 31.85; range 60–153).

There were no cases with ADHD or DLD comorbid with other clinical conditions. However, two children of the ASD group showed an ADHD condition in their reports, and a child presented ADHD and DLD.

The control group (TD) consisted of 73 parents and 73 typically developing children. The children were aged 5 to 12 years (M = 110.45 months; SD = 24.42; range 63–146), and they were matched with NDD children by gender and age (within ±3 months). In the clinical group there were 59 mothers and 14 fathers, and in the control group there were 48 mothers and 25 fathers.

Finally, children in both groups were assessed with the Raven Colored Progressive Matrices test (41) or Raven's Progressive Matrices revised version (42), depending on their age. The 73 children with NDD and the 73 children with TD were seen to present scores within 1.5 SD of the mean on these tests (NDD M percentile = 45.58, SD = 30.38; TD M percentile = 56.56, SD = 27.02). Nevertheless, no significant differences were found with Kruskal-Wallis test at the non-verbal reasoning level between the three subgroups of clinical participants when they were compared (ASD: M = 51.4; SD = 31.82; ADHD: M = 39.10; SD = 30.18; DLD: M = 41.23; SD = 26.72): H_(3)_ = 2.161, *p* = 0.339.

### Instruments

#### Contextual data on parental social support

Research group created a form where parents were asked about the following aspects: whether they attended a specific professional center for their child's problems (FS: formal support); whether they attended a family association or group (IS: informal support); whether they had more children (MCh) (other sons or daughters) and whether they had the support of grandparents for the care of their children (FamS: family support-grandparents). All the questions were dichotomous (yes or no).

#### Resilience scale (CD-RISC)

This is a questionnaire completed by families and was used to assess the resilience skills of parents of children with and without NDD.

The Resilience Scale (43) consists of 25 items with a scale from 0 to 4. The total score ranges from 0 to 100 (Tot R). The construct is composed of five factors: F1: persistence, tenacity and self-efficacy (items 10–12, 16, 17, 23–25; score 0–32); F2: control under pressure (items 6, 7, 14, 15, 18, 19, 20; score 0–24); F3: adaptability and support networks (items 1, 2, 4, 5, 8; score 0–20); F4: control and purpose (items 13, 21, 22; score 0–12); and F5: spirituality (items 3, 9; score 0–8). The authors of the questionnaire obtained good psychometric properties (43). They show that the items were grouped into five dimensions and Cronbach's alpha coefficient was 0.89; test-retest reliability, the mean (sd) CD-RISC scores at time 1 [52.7 (17.9)] and time 2 [52.8 (19.9)] demonstrated a high level of agreement, with an intraclass correlation coefficient of 0.87.

#### Structural language measure: Comprensión de Estructuras Gramaticales

The *Comprensión de Estructuras Gramaticales* (CEG) (44) is a formal measure of grammatical comprehension for children aged 4–11 years, and it is the Spanish adaptation of the Test for Reception of Grammar (TROG) (45). In the present study, it was used to assess receptive language skills of children with NDD, as measure structural language skills linked to social communication.

The CEG allows to assess children's ability to understand different types of grammatical structures that vary in lengths and degrees of complexity. The child hears a sentence and he must choose which is the one that corresponds to it between four given pictures. It contains 80 items (raw score 0–80).

The test has adequate psychometric properties: The internal consistency used as a measure of reliability (Cronbach's alpha) showed an index of 0.91; Validity of criteria, correlation values: CEG-Peabody Picture Vocabulary Test (r = 0.809, *p* < 0.001) and CEG-Illinois Test Psycholinguistic Abilities (r = 0.644, *p* < 0.001) [Peabody Picture Vocabulary Test (47)].

#### Social cognition measure: Test of emotion comprehension

The Test of Emotion Comprehension (TEC) is a formal and standardized measure of emotional understanding for children between 3 and 11 years of age which is considered an appropriate global measure of social cognition as it tests the comprehension of the nature, causes, and regulation of emotion (e.g., emotions based on external causes or in other mental states such as desires or beliefs) (45). So, it was used to obtain a social cognition measure linked with the social communication of children with NDD.

This instrument allows for the evaluation of nine components of emotional understanding: Recognition of emotions (component 1), External causes (component 2), Emotions based on desires (component 3), Emotions based on beliefs (component 4), Emotions based on memories (component 5), Regulation of emotions (component 6), Hiding emotions (component 7), Mixed emotions (component 8) and Moral Emotions (component 9). The TEC raw score ranged from 0 to 9 and it is obtained by adding the sub-scores for the nine components.

The TEC consists of 23 picture stories. A brief story is read by the examiner first, and then the child is asked to choose the correct facial expression (emotion) for the main character from among four given options. The possible emotions to appear across the 23 items are happy, sad, angry, scared and/or well.

The Spanish version of the TEC is currently under validation, so one of the authors provided the research group with the Spanish version of the instructions (translated and adapted by Carlos Hernández Blasi and Francisco Pons).

The test has good test-retest reliability after 3 months of delay [r_(18)_ = 0.84], and good test-retest correlation after 13 months of delay [r_(40)_ = 0.64 and r_(32)_ = 0.54] (48, 49). Moreover, internal consistency used as a measure of reliability (Cronbach's alpha) showed all the values were in the range of 0.61–0.97.

### Procedure

The corresponding permissions were requested from the autonomous government and the school authorities of the regular schools where the children with and without NDD attended.

At each regular school, the educational psychologist assisted the research group in recruiting the clinical sample with the proposed inclusion criteria (see next paragraph) and also age-and gender-matched peers to conform a control TD group. First, the parents of all selected children were informed of the aims of the study, and then they were given a document to sign their informed consent for their own participation and for their children's participation in the study. Finally, they were asked to fill out the informed consent form and return it to their child's teacher.

The inclusion criteria for the clinical sample were (NDD group): (1) to have a current diagnosis of ASD, ADHD or DLD according to regional county diagnostic protocols and meeting DSM-5 criteria for assessment of these disorders with specific common standardized measures; and (2) to be between the ages of 5 and 12 years. For the age-matched sample (TD group), the inclusion criteria were to be matched with a child with NDD by gender and age. In addition, children in both groups were seen to score within 1.5 SD on the Raven's Colored Progressive Matrices test (40) or with the revised version of the Raven's Progressive Matrices (41), depending on the child's age.

Each child was individually assessed by the research group during a 40-min session (approximately) in a quiet room provided by the school. The tasks were administered in random order. In parallel, parents were given an envelope containing the contextual data questionnaire on parental social support and the resilience scale for completion. They had one week to fill out the form and the questionnaire, and then give them to their child's teacher. The parent who spent the most time with the child was asked to participate.

The original sample of this study included 162 participants/parents, but 6 questionnaires were not completed by the parents, so the final sample comprised 156 participants/parents.

### Data analysis

Statistical analysis was performed using SPSS 27.0. First, the Kolmogorov-Smirnov test was performed. It was found that for the dependent variable (resilience), the data did not have a normal distribution (*p* < 0.05).

With respect to hypothesis 1, the Mann-Whitney U test was used to perform group contrasts on the level of resilience between parents of children with TD and those with NDD. To obtain the sample effect size, the statistical formula described by Tomczak and Tomczak (50) was used (r = Z/√N, where Z is taken as the absolute value; a value of 0–0.1 is considered a small effect, 0.2–0.4 a medium effect, and 0.5–1 a large effect).

In relation to hypothesis 2, the Kruskal-Wallis test was performed to analyze whether there is a difference in the level of parental resilience according to the type of NDD (ASD, ADHD or DLD). In addition, the Mann-Whitney U test was calculated to find out the difference between the three subgroups according to age, structural language (receptive grammar in this study) and social cognition (emotional comprehension in this study) performance.

Finally, with respect to hypothesis 3, the Mann-Whitney U test was performed to analyze between-group differences in the resilience of the parents of children with NDD according to the social support variables. Moreover, a hierarchical linear regression was also performed in the clinical and the three subgroups (ASD, ADHD, and DLD) to find out which factors of social support, structural language and social cognition predicted of greater development of parental resilience.

## Results

Table 1 shows the level of total resilience and the scores obtained in all the factors that make up resilience (F1: persistence, tenacity and self-efficacy; F2: control under pressure; F3: adaptability and support networks; F4: control and purpose; F5: spirituality), as well as the level of social communication (in this study, measured with grammatical and emotional comprehension) of the children in the NDD and TD groups. Between-group differences obtained with Mann-Whitney U test indicated a significant difference with a large size effect in the Total Score of Resilience (r = 0.55), finding a higher level of resilience in parents in the NDD group. Similarly, this pattern was also found in all individual resilience factors with large and medium size effects [F3 (r = 0.69), F1 (r = 0.43), F2 (r = 0.39), F4 (r = 0.28), F5 (r = 0.17)].

**Table 1:** Mean ranks and between-group comparisons between children with NDD (*n* = 73) and children with TD (*n* = 73) on Resilience Scale (Total and Factors: F1, F2, F3, F4, and F5), structural language and social cognition measures; and comparison of subgroups per separate: ASD, ADHD, and DLD.

	**NDD (*n* = 73)**	**TD (*n* = 73)**	**U**	***p***	**r**	**ASD (*n* = 36)**	**ADHD (*n* = 20)**	**DLD (n = 17)**	**H**	***p***
	**M Rank**	**M Rank**				**M Rank**	**M Rank**	**M Rank**		
**Age**	71.23	75.77	2,498.5	0.516	0.05	36.04	44.38	30.35	4.161	0.125
**Tot R**	96.53	50.47	983	0.000	0.55	42.08	37.88	25.21	7.37	0.025
*- F1*	91.64	55.36	1,340.5	0.000	0.43	40.50	39.05	27.18	4.85	0.088
*- F2*	89.97	57.03	1,462	0.000	0.39	39.67	38.33	29.79	2.62	0.269
*- F3*	102.63	44.37	538	0.000	0.69	38.51	40.35	29.85	2.72	0.257
*- F4*	85.21	61.79	1,809.5	0.001	0.28	36.39	40.95	33.65	1.17	0.557
*- F5*	80.59	66.41	2,147.0	0.040	0.17	34.39	35.83	43.91	2.46	0.291
**CEG**	53.15	93.85	1,179	0.000	0.48	31.71	52.28	30.24	14.3	0.001
**TEC**	62.99	84.01	1,897.5	0.002	0.25	31.51	50.15	33.15	11.1	0.004

Tot R, total resilience; CEG, receptive grammar (structural language measure); TEC, emotional comprehension (social cognition measure). For CEG and TEC raw scores were used.

Regarding the age of the participants, no significant differences were found between the two groups. Regarding the children's receptive grammar and emotional comprehension, significant differences were observed between the NDD and TD groups with large and medium effect sizes (CEG, r = 0.48; TEC r = 0.25).

Table 1 also shows the data of the clinical group, comparing the results obtained in each subgroup (ASD, ADHD, and DLD). In this sense, the values of the Krustal-Wallis test showed that the level of total resilience was significantly different in the three subgroups. The parents of children with ASD are the ones who obtained a higher level of resilience, followed by the ADHD subgroup, with the DLD subgroup presenting a lower level of resilience.

Between-group comparisons carried out with Mann-Whitney U test show that there were no differences in the level of resilience between parents of children with ASD and ADHD (U = 320, *p* = 0.49, r = 0.09); nor between parents of children with ADHD and DLD (U = 113, *p* = 0.082, r = 0.28); but a significant difference was found between the level of resilience of parents of children with ASD and DLD with a large effect size (U = 162.5, *p* = 0.006, r = 0.37).

With respect to the age of participants, no significant differences were observed between the three clinical subgroups.

Regarding the level of structural language and social cognition of the participants, the Kruskal-Wallis test showed significant differences in both the receptive grammar and emotional comprehension skills among the three subgroups. First, regarding receptive grammar performance, DLD subgroup underperformed ASD and ADHD groups. Between-group comparisons with Mann-Whitney U test showed that there were significant differences between the ASD and ADHD groups (U = 156, *p* < 0.001, r = 0.46), and also the ADHD and DLD group (U = 68, *p* = 0.002, r = 0.53), but not between the ASD and DLD groups (U = 292.5, *p* = 0.797, r = 0.05). Second, with respect to the emotional comprehension, ASD subgroup scored lower than the other two subgroups. In this case, Mann-Whitney U test showed that there were significant differences between the ASD and ADHD groups (U = 174, *p* = 0.001, r = 0.43), and ADHD and DLD groups (U = 92, *p* = 0.010, r = 0.40), but not between ASD and DLD groups (U = 294, *p* = 0.816, r = 0.03).

Table 2 presents data on the effect of different types of supports (formal, informal, and family) and having more children on the level of resilience of parents. Mann-Whitney U showed that there were significant differences in the level of parental resilience between those with and without formal and informal supports in the ASD group (with a medium effect size) and in the ADHD group (with a large effect size), but not in the DLD group. Related variables to family support such as having help from grandparents or having more children, did not significantly impact parental resilience in either group.

**Table 2:** Relationship between the different types of social supports and the level of resilience of parents in the different clinical groups.

	**ASD (*****n*** = **36)**				**r**	**ADHD (*****n*** = **20)**				**r**	**DLD (*****n*** = **17)**				**r**
	***n***	**M Rank**	**U**	***p***		***n***	**M Rank**	**U**	***p***		***n***	**M Rank**	**U**	***p***	
**FS**			92	0.037	0.34			18	0.017	0.53			16	0.087	0.41
No	15	14.17				10	7.35				11	7.4			
Yes	21	21.60				10	13.65				6	11.8			
**IS**			93	0.030	0.34			16	0.019	0.52			24	0.247	0.28
No	15	14.23				7	6.29				8	7.5			
Yes	21	21.55				13	12.17				9	10.3			
**FamS**			140	0.767	−0.04			27	0.153	0.31			24	0.364	−0.21
No	13	19.19				7	7.93				11	9.8			
Yes	23	18.11				13	11.88				6	7.5			
**MCh**			75	0.927	0.01			37	0.525	0.14			26	0.480	0.17
No	5	18.10				3	12.50				6	7.83			
Yes	31	18.56				17	10.15				11	9.64			

FS, formal support; IS, informal support; FamS, family support (grandparents); MCh, More children.

Finally, a hierarchical linear regression was carried out to predict parental resilience in the group of children with NDD. This analysis was performed on the total score of the Resilience Scale. To this end, five variables were used: age of children, formal support, informal support, level of receptive grammar (structural language) and level of emotional understanding (social cognition) of the children.

Table 3 shows the regression analyses of the general model obtained. A total 31.9% of the variance was explained: *F*_(4, 68)_ = 7.953, R^2^ = 0.319, *p* < 0.001. Specifically, Step 5 pointed out the importance of the variable age of children (β = 0.036) and having formal support (β = 0.003) as aspects that can predict resilience of parents with children with NDD. Nevertheless, the effect of the variables informal support, structural language and social cognition (grammar and emotional comprehension levels) were not seen significant.

**Table 3:** Summary of the regression coefficients of Resilience Scale total scores within the NDD group.

**Predictor**	**NDD (*****n*** = **73)**					
	**R^2^**	**B**	**SE B**	**β**	***t***	***p***
*Step 1*	0.029					
Constant		2.841	0.195		14.574	0.000
Age		0.003	0.002	0.171	1.460	0.149
*Step 2*	0.279					
Constant		2.590	0.177		14.653	0.000
Age		0.003	0.002	0.201	1.972	0.053
FS		0.400	0.081	0.500	4.922	0.000
*Step 3*	0.308					
Constant		2.548	0.176		14.475	0.000
Age		0.003	0.002	0.193	1.921	0.059
FS		0.318	0.094	0.398	3.400	0.001
IF		0.162	0.095	0.200	1.712	0.091
*Step 4*	0.319					
Constant		2.542	0.176		14.437	0.000
Age		0.004	0.002	0.273	2.150	0.035
FS		0.296	0.096	0.370	3.081	0.003
IS		0.180	0.096	0.222	1.869	0.066
CEG		−0.002	0.002	−0.134	−1.029	0.307
*Step 5*	0.319					
Constant		2.531	0.186		13.639	0.000
Age		0.004	0.002	0.273	2.141	0.036
FS		0.295	0.097	0.369	3.056	0.003
IS		0.180	0.097	0.221	1.852	0.068
CEG		−0.003	0.003	−0.160	−0.867	0.389
TEC		0.005	0.027	0.032	0.199	0.843

FS, formal support; IS, informal support; CEG, receptive grammar (structural language measure); TEC, emotional comprehension (social cognition measure). For CEG and TEC raw scores were used.

Similarly, three hierarchical linear regressions were carried out to predict parental resilience in each subgroup of children with NDD (ASD, DLD and ADHD separately). Again, these analyses were performed on the total score of the Resilience Scale, and the same five variables were used.

Table 4 shows the regression analyses of the general model performed for the three groups. Regarding the group of children with ASD, 27.8 % of the variance was explained: *F*_(5, 30)_ = 2.305, R^2^ = 0.278, *p* = 0.069, formal support being significant in step 2 (β = 0.42). Similarly, in the group of children with ADHD, 44 % of the variance was explained: *F*_(5, 14)_ = 2.255, R^2^ = 0.446, *p* = 0.162, with Age being significant at step 1 (β = 0.45) and formal support being significant at step 2 (β = 0.44). And finally, in the group of children with DLD, 51% of the variance was explained: *F*_(5, 11)_ = 2.268, R^2^ = 0.508, *p* = 0.120, and the significance of formal support stands out in step 2 (β = 0.60).

**Table 4:** Summary of the regression coefficients of Resilience Scale total scores within the ASD, the ADHD and the DLD group.

**Predictor**	**ASD (*****n*** = **36)**						**ADHD (*****n*** = **20)**						**DLD (*****n*** = **17)**					
	**R^2^**	**B**	**SE B**	**β**	***t***	***p***	**R^2^**	**B**	**SE B**	**β**	***t***	***p***	**R^2^**	**B**	**SE B**	**β**	***t***	***p***
*Step 1*	0.00						0.21						0.01					
Constant		3.2	0.26		12.1	0.000		2.2	0.43		5.15	0.000		2.748	0.36		7.63	0.000
Age		−9.1	0.002	−0.007	−0.03	0.970		0.008	0.004	0.45	2.17	0.044		0.001	0.00	0.10	0.393	0.700
*Step 2*	0.17						0.37						0.31					
Constant		2.97	0.263		11.3	0.000		2.40	0.403		596	0.000		2.217	0.38		5.82	0.000
Age		0.001	0.002	0.047	0.294	0.771		0.005	0.004	0.28	1.34	0.195		0.005	0.00	0.35	1.45	0.167
FS		0.301	0.114	0.421	2.64	0.013		0.342	0.160	0.44	2.13	0.047		0.526	0.21	0.60	2.44	0.028
*Step 3*	0.23						0.42						0.33					
Constant		2.87	0.264		10.9	0.000		2.18	0.444		4.92	0.000		2.216	0.38		5.71	0.000
Age		0.001	0.002	0.070	0.449	0.657		0.006	0.004	0.35	1.62	0.123		0.004	0.00	0.31	1.22	0.243
FS		0.191	0.132	0.267	1.44	0.157		0.137	0.244	0.17	0.561	0.583		0.480	0.22	0.54	2.09	0.056
IS		0.206	0.132	0.288	1.55	0.129		0.262	0.237	0.32	1.10	0.285		0.141	0.19	0.16	0.707	0.492
*Step 4*	0.26						0.44						0.35					
Constant		2.86	0.263		10.8	0.000		2.23	0.453		4.94	0.000		2.189	0.40		5.45	0.000
Age		0.003	0.003	0.179	0.980	0.335		0.009	0.005	0.52	1.73	0.104		0.002	0.00	0.15	0.370	0.718
FS		0.166	0.133	0.232	1.24	0.223		0.043	0.272	0.05	0.160	0.875		0.542	0.26	0.62	2.07	0.060
IS		0.252	0.138	0.352	1.82	0.077		0.348	0.261	0.43	1.33	0.202		0.197	0.22	0.23	0.860	0.407
CEG		−0.003	0.003	−0.212	−1.15	0.259		−0.006	0.008	−0.22	−0.82	0.424		0.005	0.00	0.25	0.547	0.594
*Step 5*	0.28						0.45						0.51					
Constant		2.91	0.276		10.5	0.000		2.10	0.860		2.45	0.028		2.240	0.36		6.12	0.000
Age		0.002	0.003	0.164	0.889	0.381		0.009	0.006	0.54	1.66	0.119		0.000	0.00	0.00	0.023	0.982
FS		0.166	0.134	0.232	1.23	0.225		0.022	0.304	0.02	0.073	0.943		0.423	0.24	0.48	1.72	0.112
IS		0.251	0.139	0.351	1.81	0.080		0.364	0.283	0.45	1.28	0.220		0.160	0.20	0.19	0.767	0.459
CEG		−0.001	0.004	−0.076	−0.29	0.770		−0.007	0.009	−0.24	−0.79	0.439		−0.009	0.01	−0.48	−0.84	0.416
TEC		−0.02	0.032	−0.174	−0.75	0.459		0.018	0.101	0.04	0.182	0.858		0.136	0.07	0.86	1.88	0.086

FS, formal support; IS, informal support; CEG, receptive grammar (structural language measure); TEC, emotional comprehension (social cognition measure). For CEG and TEC raw scores were used.

## Discussion

The present study aimed to examine the resilience of parents of children with different NDD (in this study, ASD, ADHD, and DLD), and also to examine which individual factors of the child and which contextual factors of the family support that parental resilience. So, the role of structural language, social cognition and social support of the family was explored.

With respect to Hypothesis 1, it was expected that parents of children with NDD would show higher parental resilience scores compared to parents of children with TD on all factors that make up resilience. In this sense, results of the present study verified this hypothesis and between-group comparisons pointed out that having a child with a disorder usually leads to the development of greater resilience skills, probably because these skills allow them to cope with the difficult situation and the barriers they have to face in their daily lives (41). Moreover, when analyzing the level of resilience among the different types of NDD (ASD, ADHD, and DLD per separate), between-group comparisons showed that the level of general resilience is significantly different in the different groups (*p* = 0.025). Thus, the fact of having a child with a disorder does not always mean developing greater resilience skills per se (42), but the differences on parental resilience found between the clinical subgroups [ASD-ADHD (U = 320, *p* = 0.49, r = −0.09); ADHD-DLD (U = 113, *p* = 0.082, r = −0.28); ASD-DLD (U = 162.5, *p* = 0.006, r = −0.37)] indicate that it depends on the type of disorder (42).

These results represent an important contribution to existent literature, since most of the studies assessing resilience in parents of children with NDD focus on parents with children with ASD (5–9), but a cross-disorder comparison comparing the level of resilience in parents with children with other NDD who also show social and communication difficulties were limited (51).

In this sense, it was considered necessary to analyse the difference in the level of parental resilience according to the type of disorder in order to find out the possible relationship between the type of NDD (ASD, DLD, and ADHD), the age of the children, and severity of their symptoms (taking into account their level of structural language and social cognition, as prerequisites of social communication) and the level of parental resilience. As expected in Hypothesis 2, the data from our study show that the level of resilience is greater in parents of children with ASD, followed by the group of parents of children with ADHD and the group of parents of children with DLD, the latter having the lowest level of resilience. In this sense, both ASD and DLD groups performed similarly on receptive grammar and emotional comprehension tests, but they showed significant lower scores when compared to those with ADHD. Nevertheless, total resilience scores of parents were only significantly different between these two subgroups (DLD and ASD). Thus, it seems that the lower level of receptive grammar and emotional comprehension showed by children with ASD and the level of resilience of their parents (see Table 1) is in line with previous studies, as they showed that emotional, communication and behavioral problems in children increased the mental health problems of mothers with ASD children (27).

Specifically, for children with ASD, the data of the present study shows that the lower the children's level of receptive grammar and emotional comprehension are, the higher parental resilience is. Nevertheless, this affirmation does not apply to those children with DLD and their parents. This finding may indicate that resilient skills start to develop when parents perceive their children's problems as an “adverse situation” (e.g., when a confirmed diagnose of ASD is received, in contrast to a diagnose of DLD, perhaps because it is a disorder whose prognosis and course is seen less severe. Additionally, parents of children with ASD experience a parenting dealing with greater social communication problems of their children that are present in their everyday situations (with a greater number of behavioral and emotional issues), and more severe than in children with DLD or ADHD. It seems that, during these developmental stages (school period), communicating effectively with their children, getting their children to respect social and family norms, or connect emotionally with them, can be more challenging for parents of children with ASD than for children with DLD or ADHD. Consequently, all these issues may contribute to experience the situation as more “adverse” or complex, which is a key aspect for the development of resilience (21).

Therefore, data from the present study support the results of previous studies (7, 21, 23) that associate ASD and ADHD symptoms with a higher degree of stress and mental health problems in parents, but also provide information concerning parents of children with DLD.

Finally, as stated in the first part of Hypothesis 3, it was expected to find that among personal features of children with NDD, the age and their difficulties in receptive grammar and emotional comprehension (as they are prerequisites of social communication) will predict the resilience of their parents in any type of NDD; and among contextual factors, it was expected that formal support would be the factor more associated with the resilience of parents of children with any type of NDD.

In this respect, our data partially verifies this third hypothesis. Regarding personal features of children, the age of the children did influence the resilience of the parents of children with NDD in general, although this predictive power was only observed for those children with ADHD (but not for those with ASD or DLD). Moreover, variables related to structural language and social cognition (receptive grammar and emotional understanding), were not powerful enough to predict total resilience scores of parents of children with NDD (both in general and within the three subgroups).

Furthermore, according to the second part of the Hypothesis 3, formal support was expected to be the factor that would explained more the resilience of parents of children with any type of NDD. Between-group comparisons according to the different types of social supports and the level of resilience of parents in the different clinical groups allows us to verify this assumption, pointing out the importance of having formal and informal support for the development of parents' resilience. Specially, this is important for the groups of children with ASD and ADHD whose parents showed less parental resilience (and therefore, for maybe less perceived wellbeing). Formal and informal support may provide a source of informational support (information about the characteristics of the disorder itself, as well as its evolution), instrumental support (they offer guidelines for day-to-day intervention) and emotional support (they are a source of relief and comfort) (37, 42, 46). In contrast, our data suggest that other variables like the presence of grandparents or having other children in the family (sons/daughters) do not influence the level of parental resilience. Possibly, although having support in the upbringing of children with a disorder is considered a protective factor because it relieves parents (52), sometimes the lack of understanding of some behaviors by a family member can generate more stress for parents (15). Also, several studies suggest that siblings without problems perceive their siblings with a disorder as a burden (53).

The importance of formal support was confirmed in the regression analysis, and it was found to be a significant predictor of parental residence both within the NDD group and in the clinical subgroups per separate. So, these results provide a key contribution to previous research because although many studies establish social support as a protective factor that facilitates individual resilience and reduces parental stress (54–56), most of these studies were conducted with only parents with ASD children and furthermore do not differentiate between different types of social support. Thus, formal support is also important for those parents of children with ADHD and DLD.

For example, for children with NDD and their families, one of the most effective formal support is the one offered by psycho-pedagogical counselors, as they provide the parents with guidelines adapted to the development level and real skills of the child, taking into account previously acquired knowledge and skills. In addition, they also focus on personal aspects of the child that are hindering their participation in social and academic contexts (such as reducing inappropriate behaviors); social and communication nuclear and related aspects (such as improving their structural language or social cognition skills); and academic aspects if necessary (such as improving curricular adaptations to facilitate or enable the learning process) (57).

Limitations of this study include the sample size of the three clinical groups and the unequal number of participants of the three different diagnoses (ASD, DLD, and ADHD). In this respect, future cross-disorder studies must check resilience models with similar and greater sample sizes, as the predictors of parental resilience related to structural language and social cognition may reach significance. Moreover, regarding the social cognition measure, the limited number of elements of each component and the fact that it covers partial aspects of social cognition must be noted. Maybe other features of social communication deficits are more related with resilience (e.g., pragmatics skills related to intention comprehension or dealing with complex mental states as errors or mistakes) have not been assessed with our social cognition measure (the TEC). Thus, another limitation of the present study is that no direct assessments of social communication taken in real communication settings were included. Finally, the present study focuses also on contextual aspects of parenting. So, as formal support has been seen a key factor to promote parents' resilience, another limitation of the study is that the time they have been receiving this formal support was not collected. Moreover, future studies should also focus on personal aspects of parents (such as age of parents or educational level, for example).

It must be noted that the main results of the present study have important implications for clinical and educational professionals who attend families of children with NDD. These professionals are the ones who provide formal support for parents, as mentioned above. So, when a child is diagnosed with any type of NDD, it is very important that parents are informed of the benefits of receiving this type of support.

Considering how the development of structural language and social cognition is associated with social communication skills, and how these variables are related to emotional stress (28), formal support could include counseling and training for parents in three different domains:

- in the behavioral domain, providing parents with strategies to intervene or redirect inappropriate social and emotional behaviors of their children to decrease parental stress (58);
- in the psychoeducational domain, providing parents with strategies to cope with the academic activities that children have to perform at home, and also with the socialization problems of their children (e.g., teaching them to promote social interaction of their children with their peers, where they can act as mediators; how to expand their structural language and social cognition skills with indirect strategies), which would increase parents' self-efficacy (56, 58);
- in resilience domain (that is, the development of individual skills, knowing protective factors, and knowing how to act with risk factors) (59). In this respect, the development of resilience should be worked not only with parents to reduce individual stress (60), but with the maximum family members (family resilience) to try to reduce the stress generated within the family functioning (61).

Likewise, there are other types of methods such as mindfulness-based interventions that have been shown to have a positive effect on the psychological wellbeing and stress reduction of parents with NDD (62, 63).

Another implication of the present study, addressed to autonomic governments, is the importance of reinforcing public educational centers where children with NDD attend with a greater number of educational psychologists, who could help more families to receive the formal support described. Sometimes, the professionals who attend these children do not have time to attend to all the cases they have in the school and their families, and therefore they establish a priority based on the severity of the children's symptoms.

Finally, the results also encourage families to create and/or participate in associations of relatives of children with a given disorder. These centers provide informal support to families, since they are a communication space to share experiences, a leisure space where playful activities can be carried out as a family, and also a space where training and intervention programs can be organized by professionals for families on aspects related to the child's education and from where the resilience of the family members, including the child with NDD, can be developed. With this type of support, it is intended to improve the mental health of families and therefore an improvement in their quality of life.

## Data availability statement

The datasets generated for this study are available from the corresponding author upon reasonable request.

## Ethics statement

The studies involving human participants were reviewed and approved by CD/17/2022. Written informed consent to participate in this study was provided by the participants' legal guardian/next of kin.

## Author contributions

RF-B and CA-R have contributed to the conceptualization of the study, design and development of the research, and co-directors of the research projects that funded this study. In addition, all authors have contributed to the data analysis, discussed the results, and collaborated jointly in writing the current draft and revising the final manuscript before submission.

## Funding

This study was funded by Ministry of Science and Innovation (Spain) (PID2020-115167GA-I00) and the Universitat Jaume I of Castellón (UJI-B2020-29).

## Conflict of interest

The authors declare that the research was conducted in the absence of any commercial or financial relationships that could be construed as a potential conflict of interest.

## Publisher's note

All claims expressed in this article are solely those of the authors and do not necessarily represent those of their affiliated organizations, or those of the publisher, the editors and the reviewers. Any product that may be evaluated in this article, or claim that may be made by its manufacturer, is not guaranteed or endorsed by the publisher.
